# Harmful drinking after job loss: a stronger association during the post-2008 economic crisis?

**DOI:** 10.1007/s00038-016-0936-3

**Published:** 2017-02-22

**Authors:** Moniek C. M. de Goeij, Jan-Willem Bruggink, Ferdy Otten, Anton E. Kunst

**Affiliations:** 1 Department of Public Health, J2-215, Academic Medical Center (AMC), University of Amsterdam, PO Box 22660, 1100 DD Amsterdam, The Netherlands; 2 Department of Socio-economic and Spatial Statistics, Statistics Netherlands, PO Box 4481, 6401 CZ Heerlen, The Netherlands

**Keywords:** Alcohol, Drinking, Economic crisis, Job loss, Sex

## Abstract

**Objectives:**

This study investigated, among the Dutch working population, whether job loss during the post-2008 economic crisis is associated with harmful drinking and whether this association is stronger than before the crisis.

**Methods:**

Repeated cross-sectional data from the Dutch Health Interview Survey 2004–2013 were used to define episodic drinking (≥6 glasses on 1 day ≥1/week) and chronic drinking (≥14 glasses/week for women and ≥21 for men). These data were linked to longitudinal data from tax registries, to measure the experience and duration of job loss during a 5-year working history.

**Results:**

Before the crisis, job loss experience and duration were not associated with harmful drinking. During the crisis, job loss for more than 6 months was associated with episodic drinking [OR 1.40 (95% CI 1.01; 1.94)], while current job loss was associated with chronic drinking [OR 1.43 (95% CI 1.03; 1.98)]. These associations were most clear in men and different between the pre-crisis and crisis period (*p* interaction = 0.023 and 0.035, respectively).

**Conclusions:**

The results suggest that economic crises strengthen the potential impact of job loss on harmful drinking, predominately among men.

## Introduction

Economic stressors such as unemployment are known to be related to harmful drinking. The prevalence of binge, heavy, and hazardous drinking, and alcohol abuse and dependence is generally higher among unemployed people than among those employed (Henkel 2011). To establish the direction of the relationship between unemployment and harmful drinking, some studies investigated the impact of involuntary job loss on alcohol-related outcomes. Job loss was found to increase rates of problematic drinking (Deb et al. 2011), and alcohol-related morbidity and mortality (Eliason 2014; Eliason and Storrie 2009). Further evidence for a causal impact of unemployment on drinking comes from studies that showed that a longer unemployment duration is associated with greater alcohol consumption (Frasquilho et al. 2016; Frijters et al. 2013; Garcy and Vagero 2012; Mossakowski 2008).

The prevalence and duration of involuntary job loss is increased during periods of economic shrinkage, defined as a decrease in gross domestic product (GDP), such as the global economic crisis of recent years (Department of economic and social affairs 2011). Economic crises may impact alcohol consumption through psychological distress mechanisms (de Goeij et al. 2015). According to the self-medication theory, unemployed people may respond to psychological distress by raising their levels of alcohol consumption (Bolton et al. 2009; Khantzian 1997). Several studies indeed showed that job loss increases psychological distress, which may in turn lead to more harmful drinking (Blau et al. 2013; Bobak et al. 1999; Brown and Richman 2012; Carlson 2001; Cockerham et al. 2006; Hraba et al. 2000; Kalousova and Burgard 2014). During an economic crisis, this impact may be strengthened (Aguilar-Palacio et al. 2015) because future prospects of finding a new job have become poor for those unemployed, which may be associated with higher levels of psychological distress.

**Fig. 1:**
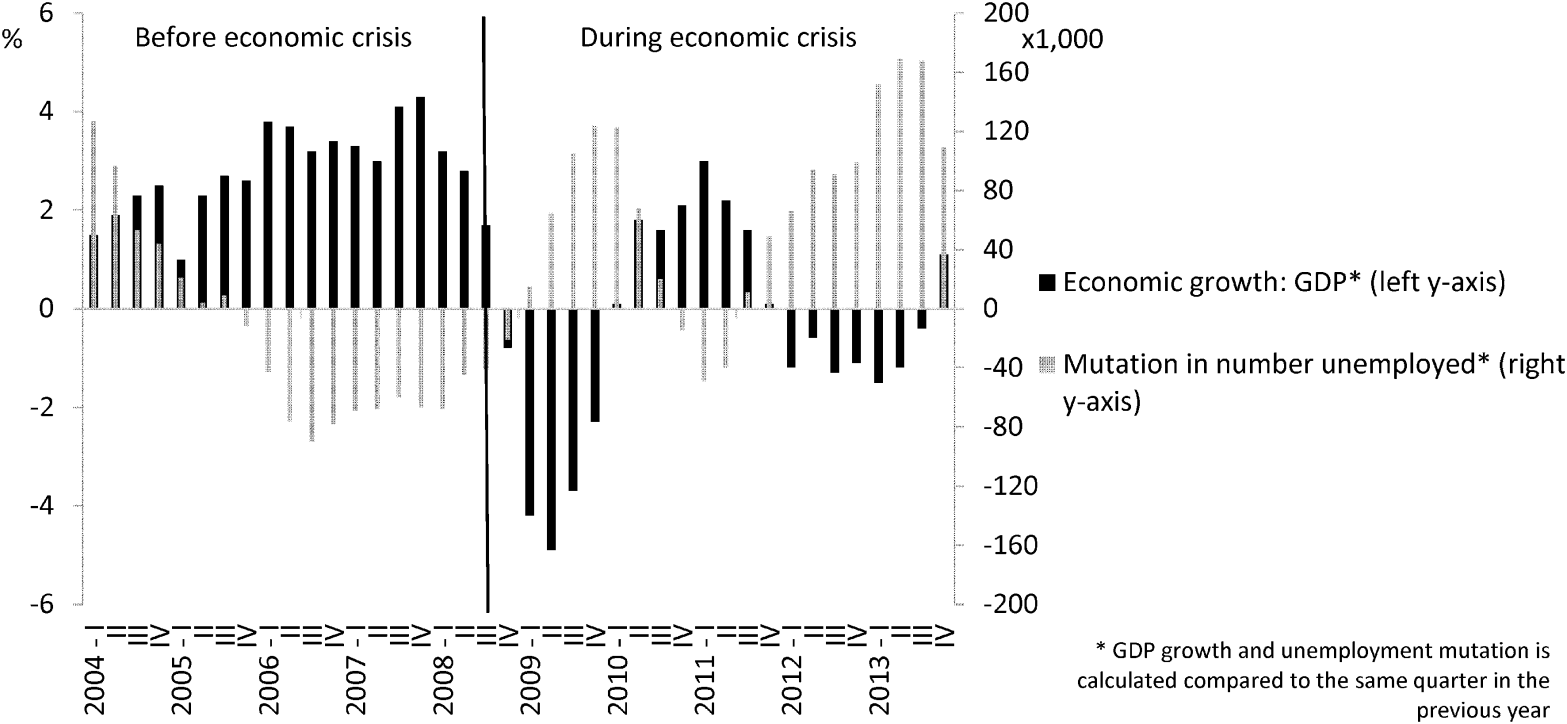
GDP growth and unemployment mutation in The Netherlands, for each quarter between 2004 and 2013

In The Netherlands, unemployment rates rose and GDP decreased during the post-2008 economic crisis (Fig. 1). According to Statistics Netherlands, the Dutch crisis started during the third quarter of 2008 (StatLine Statistics Netherlands 2015). In a previous study, we found that the post-2008 economic crisis affected the prevalence of harmful drinking in the Dutch population. More specifically, the downward trend in harmful drinking before the crisis slowed down after the crisis started (de Goeij et al. 2016). This study could not assess the mechanisms underlying this ceasing-of-decline. In principle, the impact could have arisen not only because more people had lost their job and were unemployed for a long time (volume effect), but also because the impact of job loss on harmful drinking may have increased during the crisis (intensity effect).

The current study aims to assess whether, next to the volume effect, there is evidence for an intensity effect. We specifically aimed to investigate whether the experience and duration of job loss was associated with harmful drinking during the post-2008 economic crisis in The Netherlands, and whether this association was stronger than before the crisis. These associations were studied separately for men and women, as the relation between harmful drinking and psychological distress due to economic stressors appears to be stronger among men (Bobak et al. 1999; Brown and Richman 2012; Cockerham et al. 2006).

## Methods

### Data and study population

We used data from the Health Interview Survey (HIS) 2004–2013 conducted by Statistics Netherlands. A random sample of the Dutch population living in non-institutionalized households was drawn continuously throughout the year from the Dutch Population Administration. This population was asked to fill in the first part of the HIS questionnaire, which included questions on socio-demographic variables. After completing the first part of the questionnaire, respondents were asked to also fill in the second part, which included questions on alcohol consumption. Until 2009, the first part was administered using a face-to-face interview and the second part could be filled in on paper. From 2010 onwards, a mixed-mode design was used for both the first part (via internet and in case of non-response a telephone or face-to-face interview) and second part (via internet or on paper).

The overall response rate for the first part of the HIS was approximately 60%, while response rates for the second part were approximately 50% before and 35% after the change in survey design. We used weighting factors to correct for selective non-response. Weighting factors were based on age, sex, household size, marital status, region, and (ethnic) origin. Between 2004 and 2013, a total of 117,414 respondents filled in the first part of the HIS, while the second part was filled in by 85,248 respondents.

Data for individual HIS respondents were linked to data from the Dutch tax registries on social benefits (i.e., unemployment, social assistance, disability, retirement, and other social benefits). We used month-to-month data on social benefits from the period 1999 until 2013.

For our study, we selected respondents aged 30–64 years who were part of the working-age population during the 5 years preceding the survey. To measure job loss events, respondents had to be employed at the beginning of this 5-year period and be part of the working population (i.e., employed, or involuntarily unemployed) during the following 5 years. Therefore, we excluded people without a job at the beginning of the 5-year period, those who received social assistance, retirement, disability, or other/no social benefits at any moment during these 5 years, and those who studied for at least 1 month during these 5 years. We, however, included all people who transitioned from having a job to receiving unemployment benefits, including those in which unemployment benefits were followed by receipt of social assistance benefits or not any benefit, as most of these people are likely to have had experienced an involuntary job loss. The total number of respondents aged 30–64 years was 43,642, of which 26,355 respondents remained after applying abovementioned exclusion and inclusion criteria.

### Variables

Harmful drinking was defined to include both episodic and chronic drinking. We defined episodic drinking as the consumption of ≥6 glasses of alcohol on 1 day at least once a week, for both men and women (Garretsen 1983). We defined chronic drinking as the consumption of ≥14 glasses of alcohol/week for women and ≥21 glasses of alcohol/week for men (BMA Science and Education department and the Board of Science 2008).

Job loss was defined in terms of both the experience and the duration. These concepts were measured in a person’s 5-year working history, e.g., from February 1999 until January 2004 for a person who filled in the HIS in January 2004. All people who transitioned from having a job to receiving unemployment benefits, at least once during this 5-year period, were classified as having experienced job loss. Of these people, we classified as experiencing ‘current’ job loss those who were still unemployed during the month in which they filled in the HIS (as indicated by receiving either unemployment benefits, social assistance benefits, or not any benefits). People who were re-employed by the time of survey were classified as experiencing ‘former’ job loss. We included former job loss as people with a past of job loss might retain harmful drinking patterns despite being employed again, e.g., due to increased anxiety of losing their job again. To define the duration of job loss we counted all months over a 5-year period in which a person received unemployment benefits and we added all following months in which they received either social assistance benefits or not any benefits.

We measured other variables: (1) period of survey, classified into before and during the crisis, i.e., before and after 1 September 2008, (2) age: 30–34, 35–44, 45–54, and 55–64 years, (3) country of origin: The Netherlands (i.e., both respondent, mother and father were born in The Netherlands), Western foreigner (i.e., respondent, mother, or father was born in Europe or Indonesia), non-Western foreigner (i.e., respondent, mother, or father was born in Turkey, Morocco, Dutch Antilles, or Surinam), and other countries/missing, (4) marital status: married, divorced, widowed, and never married, and (5) household composition: single, single with child(ren), couple, couple with child(ren), and single/couple with others. Country of origin was included because drinking patterns vary according to country of origin (Shield et al. 2011). Marital status and household composition were included because these factors are related to levels of alcohol consumption (Kuntsche et al. 2012; Power et al. 1999).

### Statistical analyses

As both outcome variables were dichotomous, we fitted logistic regression models. In each regression model, we included age, sex, education, country of origin, marital status, and household composition as control variables. As main variables of interest, we included period of survey, either one of the unemployment variables (i.e., job loss experience or job loss duration), and the corresponding interaction term. We estimated associations between the unemployment variable and harmful drinking conditioned on crisis, i.e., one run of the model holding crisis constant before crisis and one holding crisis constant during crisis. The interaction term was used to assess whether these associations differed between the two periods.

All models were fitted to data for the total population and separately for men and women. Weighting factors were used to take into account the selective response in such a way that the weighted sample was representative for the non-institutionalized resident population of The Netherlands for the respective survey year. Weights were applied to both the descriptions of the survey population and in the regression models reported below. We used svydesign in R to adequately adjust the standard errors for these weighting factors. Analyses were performed with SPSS/PASW version 22.0 and R version 3.02. *P* values <0.05 were considered statistically significant.

## Results

Information on episodic drinking was available for 23,812 (90.4%) respondents and on chronic drinking for 23,443 (89.0%). Table 1 presents the socio-demographic characteristics for the study sample with information on episodic drinking, also separately for men and women, before and during the post-2008 economic crisis. The majority of respondents had a Dutch origin, were married and had children. During the crisis, a larger number of people were older than 55 years, highly educated, with a non-Dutch origin, never married and single than before the crisis, both among men and women. These differences were all in line with macro-level numbers and trends in The Netherlands.

**Table 1:** Description of the study sample with available information on episodic drinking, both before (January 2004–August 2008) and during (September 2008–December 2013) the post-2008 economic crisis in The Netherlands

	All		Men		Women	
	Before economic crisis *n* = 11,189	During economic crisis *n* = 12,623	Before economic crisis *n* = 6441	During economic crisis *n* = 6659	Before economic crisis *n* = 4748	During economic crisis *n* = 5964
Age (%)						
30–34 years	14.5	12.2	13.3	11.6	16.2	13.1
35–44 years	36.2	33.4	36.0	32.7	36.5	34.1
45–54 years	33.4	34.1	33.5	34.1	33.4	34.1
55–64 years	15.9	20.3	17.3	21.6	13.9	18.7
Education (%)						
Low	24.5	23.5	24.7	24.4	24.3	22.4
Middle	42.0	37.1	41.9	35.7	42.1	38.9
High	33.1	37.0	33.1	37.6	33.1	36.2
Missing	0.3	2.4	0.2	2.4	0.4	2.4
Country of origin (%)						
The Netherlands	88.4	84.6	88.7	85.4	88.1	83.6
Western foreigner	4.2	4.2	4.0	4.2	4.4	4.2
Non-Western foreigner	2.7	4.3	2.8	3.9	2.4	4.7
Other	4.8	7.0	4.6	6.5	5.0	7.6
Marital status (%)						
Married	69.0	64.6	70.5	65.5	67.0	63.6
Divorced	8.4	10.0	6.9	8.3	10.6	12.1
Widowed	0.7	0.8	0.6	0.7	0.8	1.0
Never married	21.9	24.6	22.1	25.5	21.6	23.3
Household composition (%)						
Single	12.1	14.4	12.6	15.3	11.3	13.3
Single with child(ren)	3.9	4.6	2.1	2.6	6.4	7.1
Couple	27.4	25.6	27.2	25.5	27.8	25.7
Couple with child(ren)	56.0	54.4	57.3	55.5	54.2	53.0
Single/couple with others	0.6	0.9	0.8	1.0	0.4	0.8
Job loss experience						
Never (%)	95.8	92.9	96.0	92.4	95.7	93.4
Former (%)	2.8	4.3	2.6	4.5	3.2	3.9
Current (%)	1.3	2.9	1.4	3.0	1.2	2.7
Job loss duration						
Job loss: former						
Median (IQR), months	4 (2–10)	5 (2–10)	4 (2–11)	5 (2–10)	4 (2–8)	5 (2–10)
>6 months without job (%)^a^	33.3	39.4	38.0	41.1	27.9	36.9
Job loss: current						
Median (IQR), months	12 (6–27)	10 (5–20)	13 (7–27)	9 (5–18)	12 (4–29)	11 (5–20)
>6 months without job (%)^a^	70.5	65.4	75.5	65.4	61.9	65.4

^a^This percentage represents the proportion of respondents with either former or current job loss that have been experiencing job loss for more than 6 months

During the post-2008 economic crisis more people had experienced job loss in their 5-year working history than before the crisis: 4.3 versus 2.8% experienced former job loss, and 2.9 versus 1.3% experienced current job loss (i.e., until the time of survey). Among people experiencing former job loss, job loss duration was slightly longer during the crisis than before the crisis. Among people experiencing current job loss, this was the other way around.

**Fig. 2:**
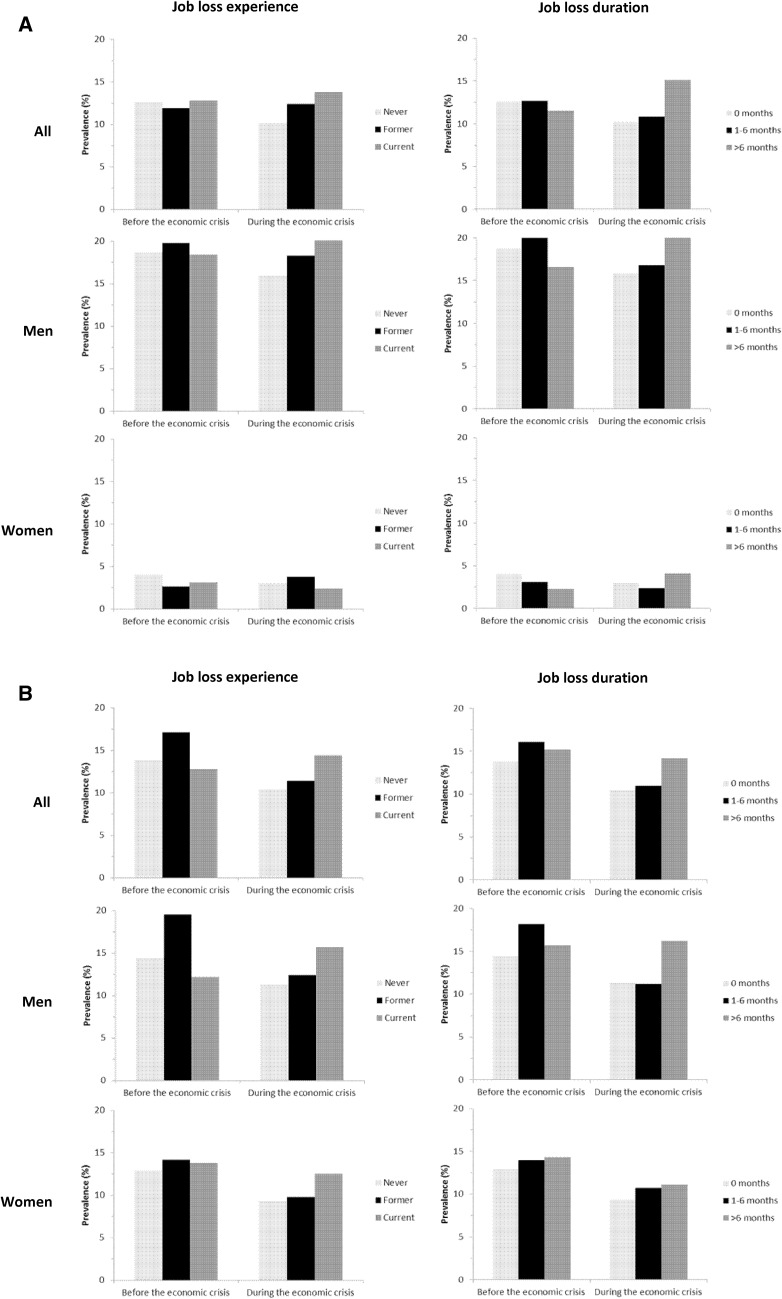
Crude levels of episodic (**a**) and chronic (**b**) drinking, both during (September 2008–December 2013) and before (January 2004–August 2008) the post-2008 economic crisis in The Netherlands

Figure 2a shows that before the post-2008 economic crisis the crude prevalence of episodic drinking is quite similar across all job loss categories. During the crisis, the crude prevalence of episodic drinking was highest among people experiencing current job loss and people experiencing job loss for more than 6 months, and lowest in those employed. The relation during the crisis with experiencing current job loss was found in men but not in women. Comparable patterns were found for chronic drinking (Fig. 2b).

The associations of job loss experience and duration with the prevalence of episodic drinking before and during the post-2008 economic crisis are presented in Table 2. Before the crisis, job loss duration was not associated with episodic drinking, while during the crisis respondents experiencing job loss for more than 6 months more often reported episodic drinking than those employed [OR 1.40 (95% CI 1.01; 1.94)]. The difference in this association between the two periods was statistically significant (*p* interaction = 0.023). This pattern was found for men (*p* interaction = 0.042) but not for women (*p* interaction = 0.274). Job loss experience (both former and current) was not associated with episodic drinking with statistical significance either before or during the post-2008 economic crisis. However, point estimates suggest a strengthening of a possible association during the crisis.

**Table 2:** Episodic drinking in relation to job loss experience and duration, both during (September 2008–December 2013) and before (January 2004–August 2008) the post-2008 economic crisis in The Netherlands

	Association before economic crisis OR (95% CI)	Association during economic crisis OR (95% CI)	*p* interaction
All			
Job loss experience			
Never	Ref	Ref	Ref
Former	0.96 (0.65; 1.41)	1.15 (0.85; 1.56)	0.461
Current	0.87 (0.53; 1.44)	1.24 (0.87; 1.77)	0.265
Job loss duration			
0 months	Ref	Ref	Ref
1–6 months	1.14 (0.76; 1.73)	0.98 (0.70; 1.37)	0.569
>6 months	0.73 (0.47; 1.15)	**1.40 (1.01; 1.94)**	**0.023**
Men			
Job loss experience			
Never	Ref	Ref	Ref
Former	1.03 (0.67; 1.58)	1.14 (0.82; 1.59)	0.704
Current	0.90 (0.53; 1.55)	1.34 (0.91; 1.98)	0.244
Job loss duration			
0 months	Ref	Ref	Ref
1–6 months	1.25 (0.79; 2.00)	1.02 (0.72; 1.47)	0.501
>6 months	0.77 (0.48; 1.25)	**1.43 (1.00; 2.04)**	**0.042**
Women			
Job loss experience			
Never	Ref	Ref	Ref
Former	0.65 (0.24; 1.80)	1.12 (0.52; 2.39)	0.409
Current	0.61 (0.14; 2.60)	0.70 (0.25; 1.97)	0.875
Job loss duration			
0 months	Ref	Ref	Ref
1–6 months	0.75 (0.27; 2.08)	0.72 (0.28; 1.82)	0.946
>6 months	0.47 (0.11; 1.99)	1.19 (0.53; 2.66)	0.274

Each of the six blocks (from “ALL/job loss experience to WOMEN/job loss duration”) is based on a separate regression model. Each model included period of survey, either one of the unemployment variables, and the corresponding interaction term. Reported associations were estimated conditioned on crisis, i.e., one model run holding crisis constant before crisis and one holding crisis constant during crisis. Age, sex, education, country of origin, marital status, and household composition were included as control variables

Bold ORs and *p*-values are statistically significant (<0.05)

Similar patterns were found for chronic drinking (Table 3). Experiencing current job loss and experiencing job loss for more than 6 months were both associated with more chronic drinking during the crisis [respectively, OR 1.43 (95% CI 1.03; 1.98), and OR 1.42 (95% CI 1.05; 1.91)]. No such association was observed before the crisis. The difference in the associations observed between the two periods was statistically significant for the experience of job loss (*p* interaction = 0.035) but not for the duration of job loss (*p* interaction = 0.144). The association of job loss duration with chronic drinking was found mostly among men. The experience of former job loss was not associated with chronic drinking with statistical significance either before or during the post-2008 economic crisis.

**Table 3:** Chronic drinking in relation to job loss experience and duration, both during (September 2008–December 2013) and before (January 2004–August 2008) the post-2008 economic crisis in The Netherlands

	Association before economic crisis OR (95% CI)	Association during economic crisis OR (95% CI)	*p* interaction
All			
Job loss experience			
Never	Ref	Ref	Ref
Former	1.36 (0.98; 1.89)	1.17 (0.88; 1.57)	0.501
Current	0.76 (0.46; 1.24)	**1.43 (1.03; 1.98)**	**0.035**
Job loss duration			
0 months	Ref	Ref	Ref
1–6 months	1.29 (0.89; 1.88)	1.14 (0.83; 1.56)	0.609
>6 months	0.98 (0.66; 1.46)	**1.42 (1.05; 1.91)**	0.144
Men			
Job loss experience			
Never	Ref	Ref	Ref
Former	1.47 (0.97; 2.22)	1.18 (0.81; 1.71)	0.430
Current	0.68 (0.36; 1.29)	1.39 (0.91; 2.13)	0.065
Job loss duration			
0 months	Ref	Ref	Ref
1–6 months	1.37 (0.84; 2.23)	1.02 (0.67; 1.57)	0.378
>6 months	0.97 (0.59; 1.58)	**1.51 (1.04; 2.20)**	0.154
Women			
Job loss experience			
Never	Ref	Ref	Ref
Former	1.23 (0.72; 2.12)	1.09 (0.68; 1.76)	0.750
Current	0.91 (0.42; 1.98)	1.39 (0.84; 2.29)	0.366
Job loss duration			
0 months	Ref	Ref	Ref
1–6 months	1.26 (0.70; 2.26)	1.36 (0.83; 2.20)	0.845
>6 months	0.96 (0.48; 1.92)	1.09 (0.67; 1.79)	0.758

See footnote to Table 2

## Discussion

### Main finding

We found that job loss duration was related to both episodic and chronic drinking during the post-2008 economic crisis, but not in the preceding years. This pattern was found predominately among men. Regarding job loss experience, we found that current job loss was related to more chronic drinking during the crisis, but not in the preceding years, while such a pattern was absent for episodic drinking. The experience of former job loss was not associated with harmful drinking, both before and during the post-2008 economic crisis.

### Evaluation of potential limitations

First, we counted job loss events during a 5-year period, which for most respondents that were interviewed during the crisis period include some pre-crisis years. Our hypothesis is that the context of the economic crisis would influence the impact of job loss on harmful drinking. Ideally, we would have measured exposure to this context for a full 5-year period. We may expect that, if this would have been possible, the association of job loss with harmful drinking as observed during the crisis would have been stronger.

Second, harmful drinking was self-reported and self-reports might underestimate alcohol consumption, especially among problem drinkers (Watson et al. 1984). Misclassifications of harmful drinking might have influenced the associations reported in our study. Though the magnitude and patterns of bias are unclear, they may not strongly differ between the two periods studied.

Third, we are one of the first to assess drinking in relationship to an individual’s working history instead of combining cross-sectional measures of unemployment and harmful drinking. The longitudinal measurement of job history supports interpretations regarding a causal relation that goes from job loss towards more harmful drinking. However, we did not include longitudinal measures of harmful drinking. As a result, it remains undetermined whether job loss experience and duration generally led to more harmful drinking, or whether harmful drinking patterns tended to precede job loss.

Fourth, Statistics Netherlands changed survey design in 2010, which may have resulted in a systematic drop or rise in the prevalence of harmful drinking. However, we were interested in the association between job loss and harmful drinking within two different time periods and not in changes over time in overall prevalence rates. In addition, a rigorous evaluation study of this change in survey design showed that it had no substantial impact on the observed prevalence of episodic heavy drinking (0.67% lower) (Wong et al. 2011).

### Interpretations

In contrast to previous studies (Deb et al. 2011; Eliason 2014; Eliason and Storrie 2009; Frijters et al. 2013; Garcy and Vagero 2012; Mossakowski 2008), we did not observe that job loss experience and duration was associated with a higher prevalence of harmful drinking before the post-2008 economic crisis. An explanation for this unexpected finding could be that in times of economic growth, job loss would generate varying mechanisms with contrasting impacts on harmful drinking. According to the literature, the main mechanisms that come into play when losing one’s job are the income mechanism (i.e., a lower income leads to less drinking) and the self-medication mechanism (i.e., psychological distress leads to more drinking) (de Goeij et al. 2015). Possibly, these two mechanisms outbalanced each other in the Dutch population before the post-2008 crisis.

Job loss duration was positively associated with harmful drinking, both episodic and chronic drinking, during the post-2008 economic crisis. A possible explanation is that in times of economic shrinkage the role of the self-medication mechanism is strengthened, unlike the income mechanism. Changes in the income effect may be limited thanks to a generous social welfare system. In The Netherlands, all unemployed people receive unemployment benefits or social assistance benefits when household income levels are below a certain threshold. The generosity of these benefits has not changed after the start of the crisis in 2008.

We thus suggest that the emergence of the association of job loss duration with harmful drinking during the post-2008 economic crisis may be due to a strengthening of the self-medication mechanism. Previous studies documented that economic crises can lead to more psychological distress, and that psychological distress can lead to more harmful drinking (Blau et al. 2013; Bobak et al. 1999; Brown and Richman 2012; Carlson 2001; Cockerham et al. 2006; Hraba et al. 2000; Kalousova and Burgard 2014). Such distress can be triggered by the stress involved in finding a new job. In times of economic shrinkage, perceived prospects of finding a new job are worse than in times of economic growth (Green et al. 2000). During crises, unemployed people may feel enhanced psychological distress already at the beginning of their job search, as they have difficulties to find a new job due to little vacancies (Clark et al. 2010). This could explain why we only found an association between current job loss, and not former job loss, with chronic drinking.

During the post-2008 economic crisis, the association of job loss duration with harmful drinking was stronger in men than in women, which is in line with the previous literature (Bobak et al. 1999; Brown and Richman 2012; Cockerham et al. 2006). An explanation for this could be that the self-medication mechanism plays a larger role among men (de Goeij et al. 2015). Men more often drink alcohol to cope with psychological distress (Bobak et al. 1999; Brown and Richman 2012; Cockerham et al. 2006), while women apply other coping strategies.

A longer duration of job loss (more than 6 months) was related to harmful drinking during the post-2008 economic crisis, but not during the preceding years. Possibly, the psychological distress that leads to harmful drinking reached detectable levels only when people were experiencing job loss for a longer period of time. This is in line with the literature demonstrating that psychological distress in unemployed people accumulates over time (Blau et al. 2013; Frijters et al. 2013; Garcy and Vagero 2012).

Current job loss was positively related to harmful drinking during the post-2008 economic crisis, while no such association was found for former job loss. A possible explanation is that people tend to change to their normal drinking pattern after finding a new job, even though some (Bolton and Rodriguez 2009) might be anxious to lose their job again. This would imply that the experience of job loss is a trigger to drink more alcohol, and re-employment would mitigate this impact on drinking patterns.

### Conclusion

The results underline that economic crises can strengthen the potential impact of job loss duration on harmful drinking. In The Netherlands, this impact became visible predominately among men, probably because of an increasing importance of the self-medication mechanism as opposed to the income mechanism. If so, this pattern of change may be particular to countries with generous welfare systems, such as The Netherlands.
